# Correlation between National Influenza Surveillance Data and Google Trends in South Korea

**DOI:** 10.1371/journal.pone.0081422

**Published:** 2013-12-05

**Authors:** Sungjin Cho, Chang Hwan Sohn, Min Woo Jo, Soo-Yong Shin, Jae Ho Lee, Seoung Mok Ryoo, Won Young Kim, Dong-Woo Seo

**Affiliations:** 1 Department of Emergency Medicine, University of Ulsan, College of Medicine, Asan Medical Center, Seoul, South Korea; 2 Department of Preventive Medicine, University of Ulsan, College of Medicine, Asan Medical Center, Seoul, South Korea; 3 Department of Biomedical Informatics, University of Ulsan, College of Medicine, Asan Medical Center, Seoul, South Korea; National Institutes of Health, United States of America

## Abstract

**Background:**

In South Korea, there is currently no syndromic surveillance system using internet search data, including Google Flu Trends. The purpose of this study was to investigate the correlation between national influenza surveillance data and Google Trends in South Korea.

**Methods:**

Our study was based on a publicly available search engine database, Google Trends, using 12 influenza-related queries, from September 9, 2007 to September 8, 2012. National surveillance data were obtained from the Korea Centers for Disease Control and Prevention (KCDC) influenza-like illness (ILI) and virologic surveillance system. Pearson's correlation coefficients were calculated to compare the national surveillance and the Google Trends data for the overall period and for 5 influenza seasons.

**Results:**

The correlation coefficient between the KCDC ILI and virologic surveillance data was 0.72 (p<0.05). The highest correlation was between the Google Trends query of H1N1 and the ILI data, with a correlation coefficient of 0.53 (p<0.05), for the overall study period. When compared with the KCDC virologic data, the Google Trends query of bird flu had the highest correlation with a correlation coefficient of 0.93 (p<0.05) in the 2010-11 season. The following queries showed a statistically significant correlation coefficient compared with ILI data for three consecutive seasons: Tamiflu (r = 0.59, 0.86, 0.90, p<0.05), new flu (r = 0.64, 0.43, 0.70, p<0.05) and flu (r = 0.68, 0.43, 0.77, p<0.05).

**Conclusions:**

In our study, we found that the Google Trends for certain queries using the survey on influenza correlated with national surveillance data in South Korea. The results of this study showed that Google Trends in the Korean language can be used as complementary data for influenza surveillance but was insufficient for the use of predictive models, such as Google Flu Trends.

## Introduction

Syndromic surveillance is defined a dynamic process of collecting real-time or near real-time data about symptom clusters that are suggestive of a biological disease outbreak[1], [2]. With international concerns about emerging infectious diseases, bioterrorism, and pandemics, the need for a real-time surveillance system has increased[3], [4]. Earlier detection will, in turn, allow for interventions that can presumably decrease the morbidity and mortality resulting from the outbreak[1], [2], [5]. Syndromic surveillance can also play an important role in monitoring the disease activity and the geographical spread of an infection, such as influenza. The 2009 (H1N1) influenza pandemic highlighted the need for a syndromic surveillance system to assist the policy and planning for effective health system responses.

Conventional surveillance for influenza is recommended to monitor influenza-like illness (ILI) and influenza virus infections. Such surveillance involves the collection and analysis of data from sentinel clinics and laboratories. Because this mode of surveillance is dependent on case reporting and medical records to track disease activity, time delays in the reporting and case confirmation can prevent early detection of outbreaks or increases in influenza. Thus, alternative data sources and real-time tools to monitor influenza are required. Alternative data sources include school absenteeism[6]–[8], over-the-counter pharmaceutical sales[9]–[11], and ambulance dispatch data[12], [13]. Using those data, disease clusters may be detected earlier than by conventional surveillance.

Recently, internet queries have been highlighted as promising data sources for influenza monitoring[14]–[18]. Every day, many users around the world search for information via web search engines. Google launched Google Flu Trends (GFT) in 2008, to estimate the national and regional influenza incidence[19]. Some studies have reported that GFT is highly correlated with conventional ILI surveillance data and that this new tool can detect regional outbreaks of influenza 7–10 days earlier than the existing surveillance system[20]–[25]. GFT has now been applied in many countries, both at a national and sub-regional level[21], [22], [25]. However, neither GFT nor other search query-based tools for disease surveillance are available in South Korea.

These search query data are available to the public using programs such as Google Trends (GT), a free service provided by Google that allows researchers to examine the trends of certain search keywords[14], [26]–[29]. This web-based service provides de-identified, normalized trend data for the search volume of certain keywords. In South Korea, there is currently no syndromic surveillance system using internet search data, including GFT. Thus, it is important to study whether this internet-based tool is feasible for influenza surveillance in South Korea. The purpose of this study was to investigate the correlation between national influenza surveillance and GT data.

## Methods

This study was approved by the Institutional Review Board of Asan Medical Center (Seoul, Korea). The study period was September 2, 2007 (week 36) through September 1, 2012 (week 35). Analyses were performed by “influenza season,” defined as the period from week 36 through week 35 of the subsequent year. Five consecutive influenza seasons (2007/08, 2008/09, 2009/10, 2010/11, 2011/12) were included. ILI and virologic surveillance data from the Korea Centers for Disease Control and Prevention (KCDC) were used to perform this analysis. We downloaded the publicly available data from the KCDC website[30]. A KCDC ILI is defined as a fever of 38°C with a cough and/or a sore throat. ILI surveillance consists of 850 sentinel clinics across the nation. The clinics report weekly percentages of outpatients who meet the case definition of ILI. The virologic surveillance data are weekly laboratory tests showing the positive rates for the influenza virus. This network consists of 91 laboratories across the nation[30].

To gather search queries related to influenza, we conducted an anonymous survey of 100 consecutive patients who visited the emergency room. The survey question was “If you've searched for influenza, what search queries or terms did you use?” Using the survey results, the definition of ILI and meetings of the authors, we picked 12 queries: new influenza (??????? in Korean), influenza (?????), new flu (????), flu (??), swine flu (????), bird flu (????), H1N1 (H1N1), bad cold (??), Tamiflu (????), fever (?), cough (??), and sore throat (???). Each query was translated into Korean. By setting the location parameter to “South Korea” and the time parameter to “2004-present”, we downloaded all these search queries from GT. Some queries that were downloaded as monthly trend data form were compared with the monthly transformed KCDC data.

Correlation analysis was performed to examine the correlation of the data from GT with the KCDC ILI and virologic surveillance data using IBM SPSS Statistics software, version 20 (IBM Corp). Strong correlation was defined as a correlation coefficient r-value of >0.7. To assess temporal relationships between GT and KCDC data for up to 2 weeks, we also performed lag correlation analysis. Significance was set at p<0.05.

## Results

**Figure 1:**
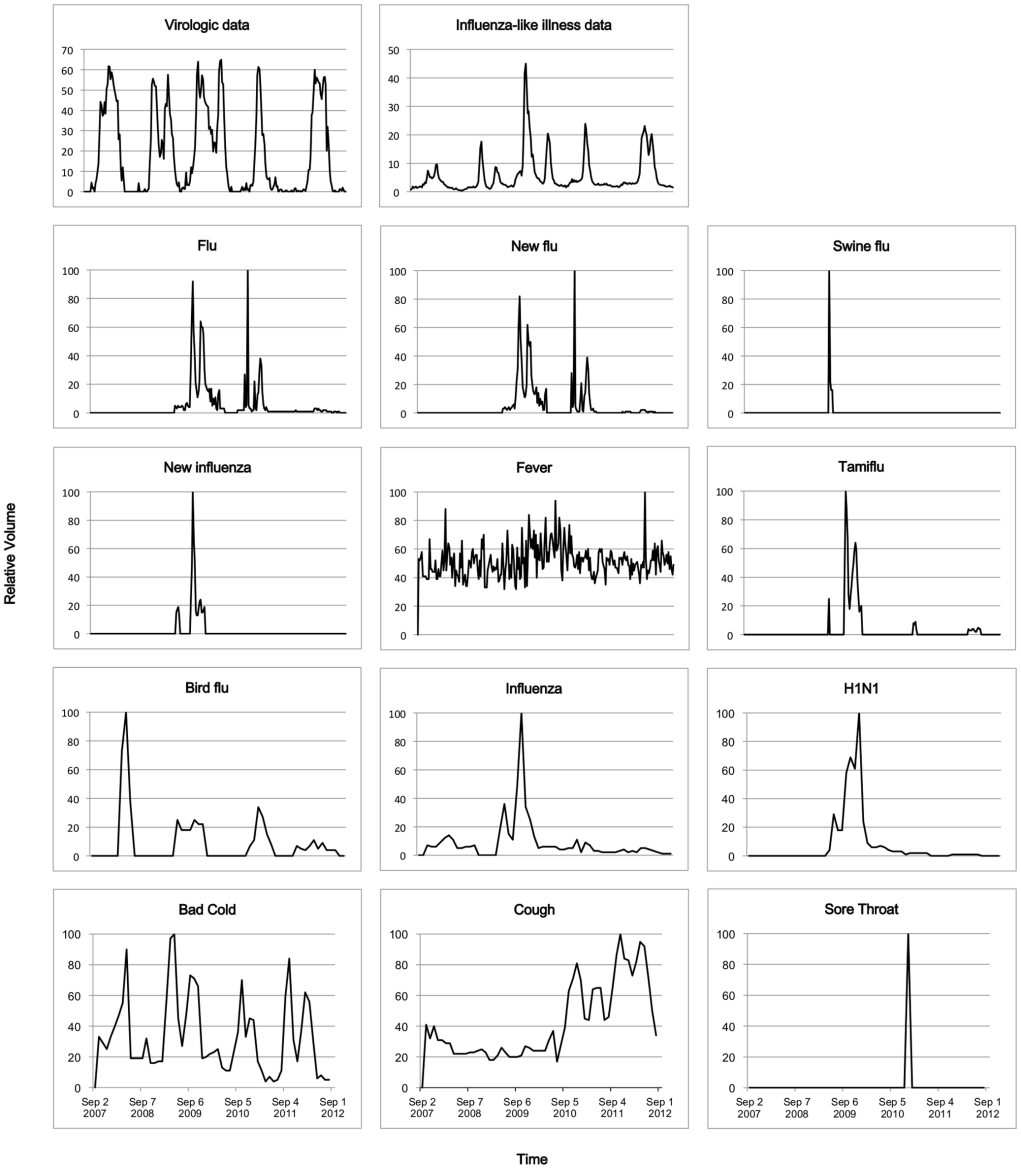
Time series plots of KCDC surveillance data and Google Trends data.

Our analyses used 254 weeks of data from the 2007/08 through the 2011/12 influenza seasons obtained from the KCDC ILI and virologic surveillance systems used to monitor national and regional influenza trends. Data included five consecutive influenza seasons, including the 2009/10 pandemic influenza season. In South Korea, each influenza season was defined as the period from week 36 through week 35 of the subsequent year. Because the weekly virologic surveillance data of the KCDC were reported from week 42 of 2007, the 2007/08 influenza season was defined from week 42 of 2007 through week 35 of 2008. The highest weekly ILI percentage was 1.0%, 1.8%, 4.5%, 2.4%, and 2.3%, chronologically, for these five consecutive years of seasonal influenza. The highest positive rate of influenza virus was 64.9%, 61.7%, 57.5%, 61.4%, and 60.0% for these years, chronologically (Figure 1).

The KCDC ILI definition of fever, cough, and sore throat was included. Bird flu and Tamiflu were added by clinicians. The GT data for the terms swine flu, new influenza, new flu, flu, fever, and Tamiflu were downloaded as weekly trend data. GT for the terms bird flu, influenza, H1N1, bad cold, cough, and sore throat were only available as monthly trend data.

The correlation between the KCDC ILI and virologic surveillance ranged from 0.72 (p<0.05) during the 2008/09 influenza season to 0.94 (p<0.05) during the 2010/11, 2011/12 influenza season (Table 1, 2). The correlation coefficient for these comparisons was 0.72 (p<0.05) for the overall study period.

**Table 1:** Pearson's correlation coefficients between Google Trends and KCDC virologic surveillance data from 2007/08 through 2011/12 influenza season.

Influenza Season	Dataset		Google Trends											
		ILI	Flu	New flu	Swine flu	New influenza	Fever	Tamiflu	Bird flu	Influenza	H1N1	Bad cold	Cough	Sore throat
2007 to 2012	Virologic	0.72*	0.15*	0.14*	0.06	−0.01	0.12	0.14*	0.09	0.07	0.19	0.33*	0.00	0.19
2007/08	Virologic	0.88*	NA	NA	NA	NA	0.33*	NA	−0.01	0.66*	NA	0.39	0.37	NA
2008/09	Virologic	0.72*	−0.22	−0.24	0.15	−0.15	−0.23	−0.16	−0.39	−0.25	−0.34	0.25	−0.04	NA
2009/10	Virologic	0.75*	0.39*	0.37*	NA	0.00	0.13	0.28*	0.18	−0.09	0.40	0.27	−0.08	NA
2010/11	Virologic	0.94*	0.35*	0.35*	NA	NA	−0.04	0.81*	0.93*	0.53	0.43	0.46	0.56	0.72*
2011/12	Virologic	0.94*	0.75*	0.68*	NA	NA	0.08	0.89*	0.59	0.78*	0.31	0.33	0.28	NA

ILI: Influenza-like illness, NA: Not applicable.

*p<0.05.

**Table 2:** Pearson's correlation coefficients between Google Trends and KCDC ILI surveillance data from 2007/08 through 2011/12 influenza season.

InfluenzaSeason	Dataset		Google Trends											
		Virologic	Flu	New flu	Swine flu	New influenza	Fever	Tamiflu	Bird flu	Influenza	H1N1	Bad cold	Cough	Sore throat
2007 to 2012	ILI	0.72*	0.44*	0.40*	0.01	0.13*	0.24*	0.40*	0.08	0.16	0.53*	0.30*	0.15	0.21
2007/08	ILI	0.88*	NA	NA	NA	NA	0.19	NA	−0.24	0.44	NA	0.21	0.40	NA
2008/09	ILI	0.72*	−0.03	−0.05	0.16	0.02	−0.37*	0.00	−0.14	0.00	−0.09	0.20	0.33	NA
2009/10	ILI	0.75*	0.68*	0.64*	NA	0.20	0.22	0.59*	0.47	0.09	0.72*	0.48	0.04	NA
2010/11	ILI	0.94*	0.43*	0.43*	NA	NA	0.01	0.86*	0.87*	0.60*	0.42	0.55	0.57	0.81*
2011/12	ILI	0.94*	0.77*	0.70*	NA	NA	0.19	0.90*	0.47	0.77*	0.28	0.42	0.28	NA

ILI: Influenza-like illness, NA: Not applicable.

*p<0.05.

The correlation between the Google Trends for 12 queries and the KCDC virologic surveillance ranged from 0.14 (p<0.05) to 0.33 (p<0.05) during the overall study period (Table 1). Four queries had statistically significant correlation coefficients, and the GT for bad cold showed the strongest correlation with the KCDC virologic surveillance during the overall study period (r = 0.33, p<0.05). The strongest correlation was between the GT for bird flu and virologic surveillance, with a correlation coefficient of 0.93 (p<0.05), during the 2010/11 influenza season. The GT for flu, Tamiflu, influenza and sore throat also had a strong correlation with the virologic surveillance (r = 0.89, 0.75, 0.78, and 0.72, respectively; p<0.05).

**Figure 2:**
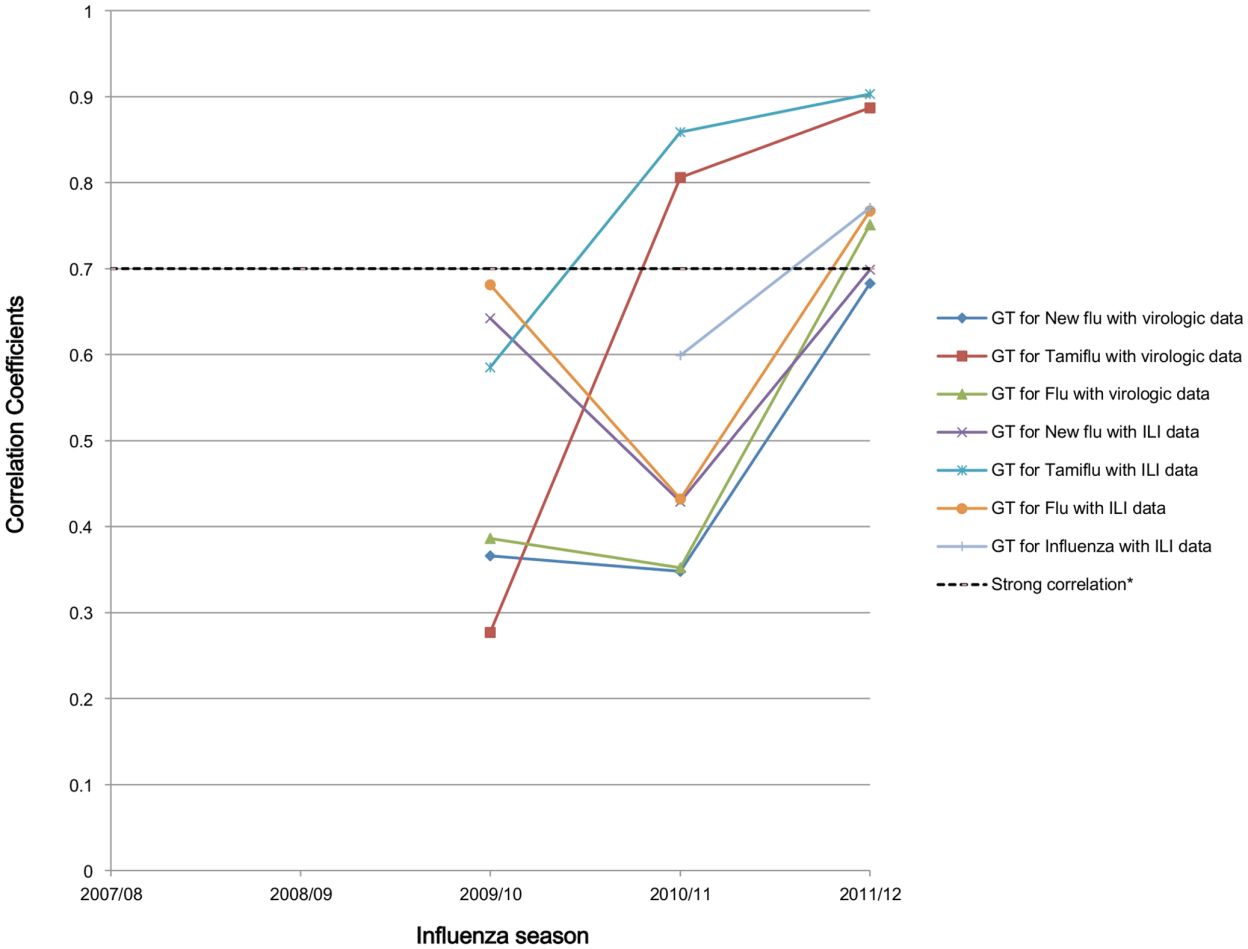
Time series plot of queries that consecutively show significant correlation coefficients (p<0.05). Strong correlation is defined as a correlation coefficient r-value of >0.7. Tamiflu is the only query to show a strong correlation for two consecutive years.

Comparisons with the KCDC ILI surveillance resulted in correlation coefficients ranging from 0.13 (p<0.05) to 0.53 (p<0.05) during overall study period (Table 2). Seven queries had statistically significant correlation coefficients, and the GT for H1N1 showed the strongest correlation with the KCDC ILI surveillance data during the overall study period (r = 0.53, p<0.05). The strongest correlation was a correlation coefficient of 0.90 (p<0.05) between the GT for Tamiflu and the ILI surveillance data during the 2011/12 influenza season. The GT for flu, new flu, bird flu, influenza and sore throat also had a strong correlation with ILI surveillance (r = 0.77, 0.70, 0.87, 0.77, and 0.81, respectively; p<0.05). Tamiflu was the only query to show a strong correlation for two consecutive years (Figure 2).

We assessed whether GT had a higher correlation with the KCDC surveillance data for influenza using lag correlation analysis (Table 3, 4). The GT data for swine flu, new influenza, new flu, flu, fever, and Tamiflu were included in this analysis, for which queries were available in the form of weekly trend data. During the study period, the correlation coefficients increased when the GT for flu, few flu, and Tamiflu were assessed against virologic surveillance data for the subsequent one or two weeks (Table 3). In the 2010/11 influenza season, the correlation between the GT for flu and new flu and the virologic surveillance increased from 0.35 to 0.38 and from 0.35 to 0.37, respectively, when assessed with a one-week lag (p<0.05). Comparing the ILI surveillance with the GT for flu, new flu, new influenza and Tamiflu showed increased correlation coefficients for the subsequent one or two weeks (Table 4). During the 2010/11 and 2011/12 influenza seasons, the GT for flu, new flu and Tamiflu showed higher correlation coefficients with a one- or two-week lag (p<0.05).

**Table 3:** Lag correlation analysis between Google Trends and KCDC virologic surveillance data from 2007/08 through 2011/12 influenza season.

Season	Dataset	Google Trends					
		Flu	New flu	Swine flu	New influenza	Fever	Tamiflu
2007∼2012	Virologic, 2-week preceding	0.08	0.06	0.11	−0.03	0.13*	0.07
	Virologic, 1-week preceding	0.13*	0.11	0.07	−0.03	0.13*	0.11
	Virologic, 0-week lagging	0.15*	0.14*	0.06	−0.01	0.12	0.14*
	Virologic, 1-week lagging	0.17*	0.15*	0.03	0.01	0.10	0.16*
	Virologic, 2-week lagging	0.17*	0.15*	0.01	0.03	0.09	0.18*
2007∼2008	Virologic, 2-week preceding	NA	NA	NA	NA	0.35*	NA
	Virologic, 1-week preceding	NA	NA	NA	NA	0.37*	NA
	Virologic, 0-week lagging	NA	NA	NA	NA	0.33*	NA
	Virologic, 1-week lagging	NA	NA	NA	NA	0.33*	NA
	Virologic, 2-week lagging	NA	NA	NA	NA	0.29*	NA
2008∼2009	Virologic, 2-week preceding	−0.24	−0.27	0.28*	−0.17	−0.27	−0.17
	Virologic, 1-week preceding	−0.23	−0.26	0.19	−0.16	−0.29*	−0.18
	Virologic, 0-week lagging	−0.22	−0.24	0.15	−0.15	−0.23	−0.16
	Virologic, 1-week lagging	−0.22	−0.24	0.08	−0.15	−0.23	−0.12
	Virologic, 2-week lagging	−0.28*	−0.29*	0.03	−0.17	−0.11	−0.07
2009∼2010	Virologic, 2-week preceding	0.30*	0.29*	NA	0.05	0.21	0.17
	Virologic, 1-week preceding	0.37*	0.34*	NA	0.01	0.15	0.27
	Virologic, 0-week lagging	0.39*	0.37*	NA	0.00	0.13	0.28*
	Virologic, 1-week lagging	0.27	0.26	NA	−0.04	0.05	0.24
	Virologic, 2-week lagging	0.24	0.21	NA	−0.03	−0.01	0.19
2010∼2011	Virologic, 2-week preceding	0.21	0.20	NA	NA	−0.32*	0.55*
	Virologic, 1-week preceding	0.32*	0.32*	NA	NA	−0.16	0.72*
	Virologic, 0-week lagging	0.35*	0.35*	NA	NA	−0.04	0.81*
	Virologic, 1-week lagging	0.38*	0.37*	NA	NA	0.02	0.75*
	Virologic, 2-week lagging	0.33*	0.33*	NA	NA	0.09	0.62*
2011∼2012	Virologic, 2-week preceding	0.68*	0.60*	NA	NA	0.20	0.80*
	Virologic, 1-week preceding	0.74*	0.64*	NA	NA	0.16	0.85*
	Virologic, 0-week lagging	0.75*	0.68*	NA	NA	0.08	0.89*
	Virologic, 1-week lagging	0.72*	0.66*	NA	NA	0.05	0.83*
	Virologic, 2-week lagging	0.67*	0.60*	NA	NA	−0.03	0.75*

NA: Not applicable.

*p<0.05.

**Table 4:** Lag correlation analysis between Google Trends and KCDC ILI surveillance data from 2007/08 through 2011/12 influenza season.

Season	Dataset	Google Trends					
		Flu	New flu	Swine flu	New influenza	Fever	Tamiflu
2007∼2012	ILI, 2-week preceding	0.24*	0.22*	0.04	0.06	0.21*	0.21*
	ILI, 1-week preceding	0.35*	0.31*	0.03	0.10	0.23*	0.31*
	ILI, 0-week lagging	0.44*	0.40*	0.01	0.13*	0.24*	0.40*
	ILI, 1-week lagging	0.47*	0.44*	0.00	0.18*	0.23*	0.46*
	ILI, 2-week lagging	0.45*	0.42*	−0.02	0.22*	0.18*	0.48*
2007∼2008	ILI, 2-week preceding	NA	NA	NA	NA	0.33*	NA
	ILI, 1-week preceding	NA	NA	NA	NA	0.23	NA
	ILI, 0-week lagging	NA	NA	NA	NA	0.19	NA
	ILI, 1-week lagging	NA	NA	NA	NA	0.19	NA
	ILI, 2-week lagging	NA	NA	NA	NA	0.09	NA
2008∼2009	ILI, 2-week preceding	−0.10	−0.12	0.27*	−0.03	−0.04	−0.08
	ILI, 1-week preceding	−0.07	−0.09	0.24	−0.01	−0.22	−0.04
	ILI, 0-week lagging	−0.03	−0.05	0.16	0.02	−0.37*	0.00
	ILI, 1-week lagging	−0.02	−0.05	0.12	0.01	−0.35*	0.04
	ILI, 2-week lagging	−0.06	−0.08	0.04	−0.03	−0.15	0.05
2009∼2010	ILI, 2-week preceding	0.43*	0.43*	NA	0.26	0.12	0.33*
	ILI, 1-week preceding	0.58*	0.55*	NA	0.24	0.18	0.50*
	ILI, 0-week lagging	0.68*	0.64*	NA	0.20	0.22	0.59*
	ILI, 1-week lagging	0.60*	0.58*	NA	0.16	0.20	0.57*
	ILI, 2-week lagging	0.51*	0.49*	NA	0.19	0.04	0.51*
2010∼2011	ILI, 2-week preceding	0.16	0.16	NA	NA	−0.29*	0.38*
	ILI, 1-week preceding	0.31*	0.30*	NA	NA	−0.16	0.68*
	ILI, 0-week lagging	0.43*	0.43*	NA	NA	0.01	0.86*
	ILI, 1-week lagging	0.44*	0.44*	NA	NA	0.10	0.87*
	ILI, 2-week lagging	0.40*	0.41*	NA	NA	0.18	0.68*
2011∼2012	ILI, 2-week preceding	0.54*	0.46*	NA	NA	0.19	0.70*
	ILI, 1-week preceding	0.67*	0.60*	NA	NA	0.17	0.81*
	ILI, 0-week lagging	0.77*	0.70*	NA	NA	0.19	0.90*
	ILI, 1-week lagging	0.81*	0.78*	NA	NA	0.17	0.93*
	ILI, 2-week lagging	0.77*	0.76*	NA	NA	0.09	0.84*

ILI: Influenza-like illness, NA: Not applicable.

*p<0.05.

## Discussion

In this study, we found that Google Trends using certain queries for influenza correlated with the national surveillance data in South Korea. To gather as many queries as possible, we conducted a survey. The survey was performed by posing a very simple question to 100 consecutive patients. We think that the results of the survey and the ILI definition (Fever, Cough, and Sore throat) represent the thinking of the public. Clinicians decided to include Tamiflu and bird flu.

Prior studies have demonstrated that internet search queries correlate with ILI or virologic in the United States and Canada[16], [18]. A study using Google AdSense[31] showed a correlation with ILI (r = 0.73, p<0.05) and virologic surveillance (r = 0.85, p<0.05)[18]. During the entire period of our study, the highest correlation coefficients were 0.33 (p<0.05) with virologic surveillance and 0.53 (p<0.05) with ILI, which were lower than those in similar studies[15], [16], [18]. However, the analysis by season showed higher correlation with the KCDC data of up to r = 0.93 (p<0.05, Table 1, 2). The GT after the 2009/10 influenza season were more strongly associated with the KCDC data than those in the prior seasons. Our study also found that the GT generally have a lower correlation with virologic surveillance than they do with ILI, which is consistent with some studies[20], [27].

In our study, Tamiflu was the only query to show a strong correlation for two consecutive years (Figure 2). Because internet search behavior may change over time, more queries that show strong correlation are required to estimate influenza outbreaks. Changing media trends, searching behavior, and regional culture may also affect the popular queries[20]. Some studies showed an estimation of an outbreak 1–2 weeks ahead of the publication of reports by each nation's influenza surveillance system[19], [29], [32]. However, Kang et al. reported no improvement in correlation with a time lag[27]. Our study found improved correlations between GT and KCDC data with time lags (Table 3, 4). This phenomenon was observed only in the 2010/11 or 2011/12 seasons. Changing search behavior due to the penetration of smartphones and the learning effect of the 2009/10 pandemic influenza season might strengthen the correlation.

There are several limitations to this study. First, although the survey is considered to represent the public, it is difficult to be sure that we selected the most relevant queries. The survey was performed after the 2011/12 influenza season. Therefore, recent search queries are likely to have been included in this study. This might have affected the outcome of this study. Second, the combination of queries and typographical errors were not included in the study. And some queries were only available in monthly form due to insufficient search volume. Third, simple correlation was used to evaluate search query data for disease surveillance in this study and GT data were provided only in the form of relative volume. Thus, the interpretation of the correlation may be affected depending on the time parameter of the GT data[33]. To minimize errors, we fixed the time parameter of the GT data. Last, news report, outbreak briefs and health publications on the internet were able to influence search behavior in a manner that did not reflect real disease activity. In this study, we did not determine the extent to which these factors affected the searching behavior.

In conclusion, we found that the GT for certain queries using the survey on influenza correlated with the national surveillance data in South Korea. The advantage of GT is that data can be obtained earlier, more easily and at little cost, whereas the published KCDC surveillance reports usually require one to two weeks for data collection and analysis. The results of this study showed that GT can be used as complementary data for influenza surveillance. However, GT was insufficient for the use of predictive models, such as Google Flu Trends. More research is required to find the most suitable queries or predictive models.
