# Correction: Correlation between National Influenza Surveillance Data and Google Trends in South Korea

**DOI:** 10.1371/annotation/4d9f12df-8b56-4e2f-b00b-4598fd5937eb

**Published:** 2014-01-23

**Authors:** Sungjin Cho, Chang Hwan Sohn, Min Woo Jo, Soo-Yong Shin, Jae Ho Lee, Seoung Mok Ryoo, Won Young Kim, Dong-Woo Seo

In the third sentence of the second paragraph of the Methods, Korean characters were inadvertently changed to questions marks during the typesetting process. The publisher apologizes for the error. The correct sentence is: "Using the survey results, the definition of ILI and meetings of the authors, we picked 12 queries: new influenza (신종인플루엔자 in Korean), influenza (인플루엔자), new flu (신종플루), flu (플루), swine flu (돼지독감), bird flu (조류독감), H1N1 (H1N1), bad cold (독감), Tamiflu (타미플루), fever (열), cough (기침), and sore throat (인후통)."

